# Common structure in the heterogeneity of plant-matter decay

**DOI:** 10.1098/rsif.2012.0122

**Published:** 2012-04-25

**Authors:** David C. Forney, Daniel H. Rothman

**Affiliations:** 1 Lorenz Center and Department of Earth, Atmospheric, and Planetary Sciences, Massachusetts Institute of Technology, Cambridge, MA 02139, USA; 2 Department of Mechanical Engineering, Massachusetts Institute of Technology, Cambridge, MA 02139, USA

**Keywords:** carbon cycle, respiration, disordered kinetics, litter decomposition

## Abstract

Carbon removed from the atmosphere by photosynthesis is released back by respiration. Although some organic carbon is degraded quickly, older carbon persists; consequently carbon stocks are much larger than predicted by initial decomposition rates. This disparity can be traced to a wide range of first-order decay-rate constants, but the rate distributions and the mechanisms that determine them are unknown. Here, we pose and solve an inverse problem to find the rate distributions corresponding to the decomposition of plant matter throughout North America. We find that rate distributions are lognormal, with a mean and variance that depend on climatic conditions and substrate. Changes in temperature and precipitation scale all rates similarly, whereas the initial substrate composition sets the time scale of faster rates. These findings probably result from the interplay of stochastic processes and biochemical kinetics, suggesting that the intrinsic variability of decomposers, substrate and environment results in a predictable distribution of rates. Within this framework, turnover times increase exponentially with the kinetic heterogeneity of rates, thereby providing a theoretical expression for the persistence of recalcitrant organic carbon in the natural environment.

## 1. Introduction

Greater than 90 per cent of the carbon dioxide input to the atmosphere–ocean system each year derives from the natural decay of organic carbon [1,2]. Decay is heterogeneous in space and time: organic molecules vary in lability [3,4]; micro-environmental heterogeneity such as the aggregation of minerals in soil and sediments interfere with degradation [5]; humification and repolymerization [4,6] result in polymers that are difficult to degrade; and decomposer communities are diverse and varied [4,7]. Physical and chemical changes in local environment [8] can speed up or prevent decomposition altogether. Spatial heterogeneity of soil nutrient concentrations at the meter scale [9] may also influence degradation rate heterogeneity. Combined, these diverse effects yield kinetic heterogeneity: older compounds appear to decay at slower rates than younger compounds [10,11], and carbon stores and their turnover times are larger than predicted from initial decomposition rates [12].

The sizes of the organic carbon stores and their rates of turnover are required for quantifying feedback between climate and the carbon cycle [13,14] in order to predict changes in carbon dioxide levels and climate [1,2,8,15]. Because decay time scales vary widely, from minutes to millions of years, estimates of carbon stocks and turnover times require knowledge of all decay rates, from fast to slow [16,17]. However, rate distributions and the mechanisms that determine them remain unknown. Identification of rate distributions should provide insight, not only for predictive purposes [17–19] but also for understanding the ecological dynamics [20] of decomposition.

Because the wide range of time scales makes it impossible to directly measure decay over all phases of decomposition, we focus on plant litter decay and early transformations to young soil organic matter. Specifically, we investigate measurements from the Long Term Intersite Decomposition Experiment Team (LIDET) study [2,12,21–23]. This study monitored the decomposition of 27 different types of litter, including needles, leaves, roots, wood, grass and wheat distributed among 28 different locations across North America ranging from Alaskan tundra to Panamanian rainforests. Litter was collected and then re-distributed in litter bags at different sites in order to investigate the effect of composition, ecosystem and climatic parameters on decomposition. Litter bags were collected and analysed at least once per year for up to 10 years. We show how to estimate kinetic heterogeneity from these observations of decay. We also analyse how these rate distributions are related to climatic conditions and litter composition.

The remainder of this paper is organized as follows. In §2, we pose and solve an inverse problem to find the rate distributions corresponding to the decomposition of plant matter from the LIDET study. We then show in §3 that the distributions are lognormal on average. Subsequently, in §4, we show how the two parameters of the lognormal distribution depend on composition and environment. In §5, we derive the relation of these parameters to the turnover time of carbon stocks. These results show that turnover times grow exponentially as the heterogeneity of rates increase, thereby highlighting the dependence of carbon stocks on their slowest rates of decay [17].

## 2. Disordered kinetics

Organic matter decomposition may be viewed as the relaxation of a kinetically disordered system. In this section, we specify a model for the influence of disorder on decay. We then describe how we invert it to obtain a distribution of decay rates.

### 2.1. Model

We suppose that decay rate constants *k* derive from stochastic reactions between heterogeneous substrates and ecological communities in a random environment. We describe this scenario using a ‘static’ model of ‘disordered kinetics’ [24–26]. In this model, the mass *g*(*t*) is a decreasing function of time that derives from a continuous superposition of exponential decays e^−*kt*^ weighted by the probability 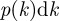 that the rate constant *k* is present at the onset of decay. Given these assumptions, decay proceeds as2.1
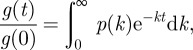
where *p*(*k*) ≥ 0 and 
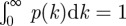
. Models similar or identical to equation (2.1) have been previously employed to describe organic matter decay [17–19,27–33]. When the distribution *p*(*k*) is discrete, the integral in equation (2.1) becomes a sum known as a ‘multi-G’ or ‘multi-pool’ model [21,34,35]. Although equation (2.1) lacks detailed mechanisms of the processes involved in decomposition, its simplicity and common application suggest that it is a reasonable first attempt at characterizing decomposition dynamics. Dispersion of the rates *k* in this model are probably associated with variations in the quality of plant-matter compounds [36,37], which range from highly labile simple sugars to more refractory lignin, waxes and phenolic compounds [35]; local spatial heterogeneity in soil moisture and nutrients [9]; chemical transformation of compounds [6] and decomposer and metabolic diversity [38,39]. Rather than attempting a detailed characterization of these individual mechanisms, we simply seek the distribution of rates associated with the minimal description of decomposition given by equation (2.1).

Although equation (2.1) represents a system of parallel steady decays, decomposition also involves temporal disturbances and serial processes. However, serial transformation processes can be mathematically expressed as parallel decays [17]. Regarding temporal fluctuations, we interpret the steady distribution *p*(*k*) as the probability that decay occurs at an effective first-order rate *k* that is averaged over seasonal and other disturbances [17]. We also note that if the difference between the time scales of two serial processes is large, the system effectively relaxes at the time scale of the slower process. For example, the degradation time scale of a particle attached to a mineral surface may be much larger than the duration of the transient period before attachment; similarly, the time scale of humification is probably short relative to the lifetime of the slowly degrading humic substance [17]. Decomposition may be approximated as proceeding initially from the mineral-associated or humic state [17]. A consequence of a parallel decay model is that resulting decays *g*(*t*) are convex (concave-up). Specifically, any completely monotone decay *g*(*t*)/*g*(0) can be described by a linear superposition of rates weighted by a probability density function *p*(*k*) [40].

We also note that the ‘random rate model’ [24] represented by equation (2.1) has been commonly used to solve problems involving heterogeneous relaxation in other fields. Examples include nuclear magnetic resonance (NMR) spin decay [41,42], protein state relaxation [43], as well as dielectric, luminescent and mechanical relaxations [24–26].

### 2.2. Inverse problem

Under certain physical conditions, distributions *p*(*k*) of reaction rates can be calculated analytically [24,27,28] and evaluated by comparing *g*(*t*) to experimental data. However, given the complex nature of decomposition, purely physical models may not be appropriate. We therefore seek to identify the distribution that best fits the data without resorting to assumptions beyond those implied by equation (2.1). Once the best distribution is found, physical reasoning then allows the identification of mechanisms that can generate this distribution.

Mathematically, equation (2.1) is a Laplace transform and *p*(*k*) can be found from its inverse. However, the inverse Laplace transform is ill-posed [44], meaning that small changes in the data *g*(*t*) can result in large changes in the solution *p*(*k*). A standard method to solve such ill-posed problems is to seek solutions *p*(*k*) that are minimally ‘rough’ [44]. Here, we use Tikhonov regularization [44,45] to identify an optimally smooth *p*(*k*) that best fits the data (§2). Such methods have been previously applied to problems of NMR spin relaxation [41] to probe the structure of porous media [46,47] and the properties of biological tissue [42].

## 3. Rates are distributed lognormally

**Figure 1.**
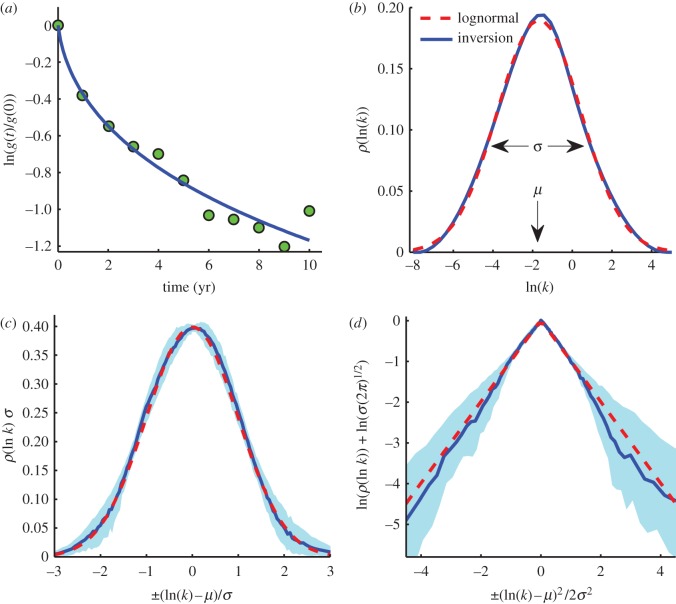
Rate distributions of plant-matter decay. (*a*) Litter decay from a LIDET dataset. Circles are data points. The curve is the predicted decay corresponding to the forward Laplace transform of the solid (blue) curve in (*b*). (*b*) Solid curve (blue) is the solution *ρ*(ln *k*) to the regularized inverse problem. Dashed curve (red) is a Gaussian distribution fit to *ρ*(ln *k*). *σ*^2^ is the variance of the Gaussian and *μ* is its mean. (*c*) (*b*) shows just one inversion, whereas the solid curve (blue) is the average of the 182 solutions *ρ*(ln *k*) having non-zero variance, each rescaled by the dataset-dependent parameters *μ* and *σ*. Dashed curve (red) is a Gaussian with zero mean and unit variance. The shaded area contains the middle 68% of the numerical inversion results. (*d*) Logarithmic transformation of the results of (*c*), where the dashed (red) straight lines indicate an exact lognormal distribution.

We apply this procedure to litter decomposition data from the LIDET study. An example of decay from an LIDET dataset is shown in figure 1*a*. The corresponding estimate of the rate distribution in logarithmic space, expressed as 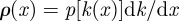, where *x* = ln *k*, is shown in figure 1*b*. The rate *k* is rescaled by the period of seasonal forcing (1 year) and is therefore non-dimensional. The good fit of *ρ*(ln *k*) to a Gaussian indicates that the distribution of rates is lognormal, characterized by the parameters *μ* and *σ*, where *μ* is the mean of ln *k* and *σ*^2^ is the variance of ln *k*.

To investigate the extent to which the lognormal distribution applies to the remainder of the LIDET data, we identify the 234 LIDET datasets that contain at least five measurements with replicates. These datasets contain 11 different litter types distributed among 26 sites. We then employ several tests on each of these 234 datasets to check whether equation (2.1) is an appropriate description of these datasets. First, we find that seven of these datasets show insignificant mass loss between the first and last field measurement, rendering equation (2.1) irrelevant. Six of these datasets are associated with root decay, suggesting that roots can persist for long times in certain conditions.

We next identify the datasets that decay faster than exponentially, counter to the assumption of first-order kinetics and a superposition of exponential decays. We use two tests to identify these datasets. First, we check the curvature of the datasets and find that nine of the datasets have negative curvature (concave-down). Such superexponential decay cannot be consistent with the Laplace transform relation (2.1) [40]. These datasets are primarily located at sites having low precipitation, indicating that decay dynamics may be limited by moisture or decomposer activity, rather than substrate availability. Second, we apply our inversion procedure to the remaining datasets and find that three datasets have a significant trend in the residual error. These datasets decay faster than exponentially, but are not concave down. All three of these datasets are associated with wood decay. In summary, our tests disqualify 7 + 9 + 3 = 19 datasets from further consideration. Further details of the tests are given in appendix A.1.

Of the remaining 215 datasets, our inversion procedure indicates that 33 datasets are characterized by a single rate constant and decay exponentially. Guided by the result of figure 1*b*, we then fit a Gaussian to the 182 estimates of *ρ*(ln *k*) exhibiting a non-zero variance and rescale each by the fitted parameters *μ* and *σ*. We plot the mean of the rescaled distributions in figure 1*c*. Although there is scatter and skew among the individual estimates of *ρ*(ln *k*), figure 1*c* shows that the mean of the rescaled distributions of ln *k* is very similar to a Gaussian distribution. Because the 33 single-rate datasets correspond to a lognormal distribution with zero variance, our results indicate that the lognormal represents the average rate distribution of the 215 datasets for which the model (2.1) applies.

Lognormally distributed variables arise naturally from multiplicative stochastic processes [48]. Here, lognormally distributed rates may result from the multitude of seemingly stochastic requirements for decomposition, such as the presence of water, the presence of an appropriate microbe, the lack of predation, the conditions for expression of hydrolytic enzymes, the encounter of enzymes with the organic matter, etc. [6]. More generally, the probability of completing any task that relies on the successful completion of many subtasks is lognormal [49]. In this context, the lognormal can be viewed as a null hypothesis in which decomposition rates result from the occurrence of a large number of independent decay requirements [6]. Mathematically, if we assume that the probability *P* of decomposing a parcel of organic matter over a time span *Δt* is the product of independent probabilities of satisfying various requirements for decay over that interval, then the first-order rate constant *k* = *P*/*Δt* becomes asymptotically lognormal as the number of requirements increases. In this manner, the multiplicative stochasticity of a decay system results in the lognormal distribution. This general description suggests that attempts to precisely model the individual mechanisms that stochastically interact to form this broader pattern would be overly complex. It also agrees with the idea that decay rates are the product of many compositional and environmental effects [22].

Previously suggested forms of the rate distribution *p*(*k*) are the gamma distribution [17,27] and the log-uniform distribution [28]. The log-uniform distribution, for which *ρ*(ln *k*) is constant and 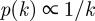 between prescribed limits, approximates the lognormal when 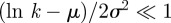 [49]. Moreover, its Laplace transform asymptotically approaches the Laplace transform of the lognormal distribution as 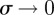. The gamma distribution, however, differs significantly from the lognormal. We find that the lognormal distribution predicts both the data *g*(*t*) and describes the inferred rate distribution *p*(*k*) better than the gamma distribution for 177 out of 215 datasets (electronic supplementary material, §2). We have also compared the lognormal to exponential and multi-pool models. Because our inversion procedure indicates that only 33 of 234 datasets are described by a simple exponential decay, we find that simple exponential decays are generally under-parametrized for describing litter decay datasets, consistent with previous studies [12,35]. The best fitting type of multiple pool model varies widely among the datasets, with no single model type describing all datasets [12,50]. A universal multiple pool model, containing pools of various types (leached, labile, refractory, inert, etc.), would be over-parametrized. Furthermore, the number of pools and rates associated with each pool are sensitive to noise, as different combinations of pools can represent the same decay [51,50]. This sensitivity makes understanding the constitutive relationships between pools and environmental and compositional parameters difficult [31].

An advantage of the lognormal is that it parametrizes decay by only two variables, *μ* and *σ*. We proceed in §4 to identify relations between the lognormal parameters *μ* and *σ*, and the climatic and compositional parameters associated with the LIDET study.

## 4. Controls on the lognormal parameters

We seek an understanding of the controls on *μ* (the mean order of magnitude of rates) and *σ* ^2^ (the variance of those orders of magnitude). Before analysing all 215 estimates of these parameters, we identify values of *μ* and *σ* that are highly uncertain by disregarding the small fraction of datasets having anomalously long turnover times *τ*. Assuming a soil carbon store is in steady state with a constant litter input, its turnover time *τ* is equal to its mean residence time [52], which in the random-rate model (2.1) equals the mean time constant 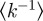 [31]; thus4.1
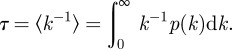


After evaluating the turnover times associated with all 215 datasets using equation (4.1), we find that there is a distinct group of datasets associated with excessively long turnover times greater than 1000 years (figure 5). These datasets contain a significant mass fraction that is effectively inert, having unknown decay dynamics. Extrapolating the kinetics of such slow processes therefore has considerable uncertainty. There are 24 datasets in this outlying cluster. These data are typically associated with root decay at certain locations (table 2), suggesting that the soils of certain ecosystems can enable the persistence of roots for long times. We do not consider these 24 datasets in our subsequent analysis of *μ* and *σ*. Further discussion of these outliers can be found in appendix A.1.

Owing to the nature of the LIDET study, many different litter types were placed at the same site and we therefore have many estimates of *μ* and *σ* at each value of temperature, precipitation and other climatic variables. Similarly, because each litter type was planted at many sites, there are many different estimates of *μ* and *σ* for each value of initial lignin concentration, nitrogen concentration, etc. In the following section, we study how the average values 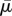 and 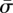 of the lognormal parameters *μ* and *σ* vary with measured independent variables such as temperature, lignin, nitrogen, etc. When analysing the effects of climatic variables, 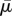 and 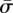 represent the averages over all litters at each site, and when analysing the effects of compositional variables, 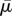 and 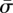 represent the averages over all sites where the litter was deployed. Similarly, 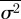 represents the average variance 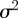, and 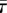 represents the average turnover time, etc. Analagous depictions of the unaveraged data can be found in appendix A.3.

### 4.1. The mean µ

**Figure 2.**
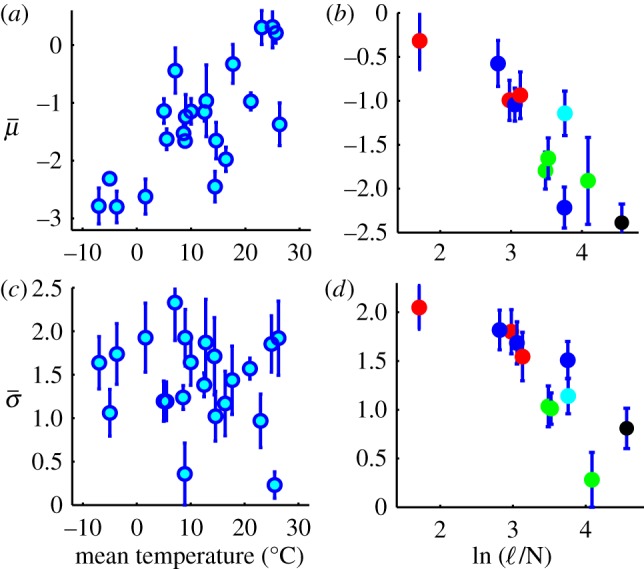
Plots of the lognormal parameters 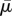 and *σ* versus experimental variables. (*a*) 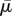 versus mean annual temperature. The Spearman rank-correlation coefficient *r*_s_ indicates a significant positive trend (*r*_s_ = 0.62, *p* = 0.002, *n* = 22). (*b*) 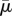 versus the initial litter lignin-to-nitrogen ratio ℓ/N (*r*_s_ = 0.89, *p* = 0.004, *n* = 11). (*c*) 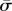 versus mean annual temperature shows no significant relation (*r*_s_ = −0.13, *p* = 0.56, *n* = 22). (*d*) 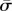 versus ℓ/N (*r*_s_ = 0.92, *p* < 10^−5^, *n* = 11). The colour of data points in panels (*b*,*d*) indicates tissue type: roots (blue), leaves (red), needles (green), wood (black) and wheat (cyan). The data in (*a*,*c*) represent 22 sites containing at least six different litters each, while the data in (*b*,*d*) represent 11 different litter types planted in at least four different locations. Error bars represent one s.d. of the mean.

We first investigate how climatic conditions and composition affect 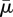. Figure 2*a* shows a significant positive correlation between 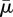 and temperature. From this trend, we find that the median decomposition rate 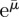 increases by a factor *Q*_10_ = 2.0 ± 0.3 (1 s.d.) with a 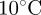 increase in temperature, in agreement with previous estimates [2]. All other measured and synthetic climatic parameters also significantly correlate with 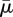, with the climate decomposition index [21,22] exhibiting the highest correlation (table 1).

**Table 1.** Spearman rank correlation coefficients *r*_s_ of field experiment parameters versus 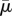 (left columns) and 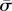 (right columns). (*p*-values are based on number *n* of samples used in the rank correlation (final column).)

	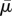		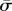		
parameters	*r*_s_	*p*	*r*_s_	*p*	*n*
precipitation	0.63	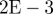	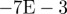	0.93	22
temperature	0.62	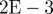	−0.13	0.56	22
latitude	−0.51	0.02	0.11	0.62	22
actual evapo-transpiration	0.72	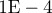	0.11	0.62	22
potential evapo-transpiration	0.42	0.05	−0.18	0.41	22
climate decomposition index [21]	0.88	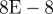	−0.02	0.88	22
C/S	−0.71	0.02	−0.87	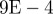	11
C/N	−0.77	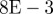	−0.85	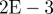	11
C/P	−0.45	0.17	−0.48	0.14	11
K	0.65	0.03	0.55	0.09	11
lignin	−0.78	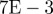	−0.71	0.02	11
lignin/N	−0.89	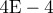	−0.92	0	11
ash	0.68	0.03	0.75	0.01	11
metal	0.63	0.04	0.43	0.18	11
tannin	0.36	0.27	0.25	0.45	11
water soluble	0.52	0.1	0.47	0.15	11
water soluble carbohydrate	0.32	0.34	0.35	0.30	11
cellulose	−0.39	0.24	−0.41	0.22	11
non-polar extractive	−0.23	0.50	−0.37	0.26	11

The parameter *μ* is also related to composition: figure 2*b* shows that 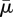 decreases as the initial lignin-to-nitrogen ratio (ℓ/N) increases. The observed trend indicates that increases in the lignin concentration, a refractory component of plant matter, are associated with a reduction in 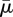, while increases in organic nitrogen, an important nutrient for microbial decomposers [36,37], are associated with an increase in 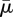. This is consistent with the use of ℓ/N as a measurement of litter quality [6,21,22,53]. The carbon-to-nitrogen ratio (C/N) and other nutrient measures are also correlated with 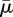 (table 1). Concentrations of lignin, N, S, P, etc., represent initial values associated with each type of litter. Figure 2*b* also indicates that needles have lower values of 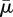 than leaves. This effect, however, may be related to the difference in ℓ/N between the two tissue types.

### 4.2. The variance σ^2^

We next investigate the relation of climatic conditions to the heterogeneity of decomposition rates, represented by *σ*. Figure 2*c* shows that temperature has no significant effect on 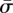. Moreover, 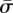 is uncorrelated with all climatic parameters monitored in the LIDET study (table 1); thus climatic conditions appear unrelated to *σ*. We therefore find no evidence from the decadal LIDET data that the *Q*_10_ of refractory components is significantly different than the *Q*_10_ of labile components. This supports respiration models such as CENTURY [16,54], which uses the same temperature and soil moisture factor for each pool of organic matter, independent of lability. We note that if rate dispersion reflects the variation in activation energies of decay processes [55], then Arrhenius kinetics suggest that *σ* only slightly decreases with temperature over the 35° temperature range associated with the LIDET sites. This is consistent with the data presented in figure 2*c*, but the wide variation in 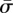 indicates that this trend is not significant and that kinetic heterogeneity is controlled by other variables.

Although 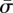 exhibits no relation to climate, it does vary with composition. Figure 2*d* indicates that 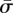 decreases as the initial lignin-to-nitrogen ratio (ℓ/N) increases. Because ℓ/N correlates negatively with 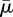, decreasing the ratio of these components tends to both shift and stretch the rate distribution, increasing the rate constants *k* of the faster decay processes, while the rate constants of slower, more refractory processes are relatively unchanged. Nutrients such as N and S, and to a lesser extent P and K, exhibit similar relationships with 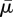 and 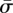 (table 1). Physically, these relationships indicate that nutrient limitation is present at early times as faster processes appear to depend strongly on the nutrient content of the litter. Slower, more refractory processes take place at rates probably sustained by the transport and immobilization of nutrients from the surrounding soil [53] and are not nutrient-limited. In fact, increased nitrogen content may inhibit the degradation of transformed plant compounds [6], widening the slow tail of the distribution and increasing *σ*. Lignin, on the other hand, may reduce the rate constants *k* of more labile compounds by shielding them via a ligno-cellulose polymer matrix [21], suggesting that ℓ/N measures a resistivity to initial decay. The effect of ℓ/N on 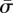 also appears to saturate at low ℓ/N, suggesting that these mechanisms lose control after crossing a threshold [6] of high N or low lignin content is reached. We also observe in figure 2*d* that roots and leaves tend to have higher 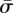 than needles, yet the effect of ℓ/N on 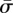 appears less strong for roots and wood, both of which decompose underground. Roots and wood do however follow the trend of 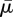 versus ℓ/N, suggesting that the effect of initial composition may persist over time in roots and wood, effecting a wider portion of their rate distribution, not just the fast rates. This behaviour may be related to components in their tissues, underground decomposition or both.

Collectively, the results of figure 2 and table 1 suggest that climate variability changes the median rate of decay, e^*μ*^, whereas the variance of decay time scales, *σ*^2^, appears to be a property of the litter sample itself and its relationship to the decomposer community inhabiting it.

### 4.3. Further trends

Table 1 identifies additional correlations between climatic, compositional and the lognormal parameters. Sulphur, another important microbial nutrient, is highly correlated with both 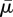 and 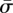. Potassium exhibits a similar trend as well. The causality of the trends in table 1 however is not always clear. For example, ash also has a significant positive correlation with both 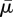 and 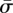. However, this is most probably explained by the strong rank correlation (*r* = 0.89) between ash and sulphur, as well as a strong correlation between ash and metals which also have a positive correlation with 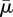 and weak correlation with 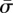. Ash is composed of sulphates, K, P, Ca and other metals [4]. Phosphorus surprisingly does not show as strong a signal as N or S and its large *p*-values suggest that trends with 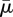 and 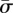 may not be significant. It is possible that the initial phosphorus concentrations may contain errors because phosphorous, as with N, S, K and Ca, is present in lower concentrations in conifer needles than in deciduous leaves [56]; the values of phosphorus measured in the needles and leaves of the LIDET study do not follow this pattern. Metals contain some important rare nutrients for microbial decomposers; we find that they are more significantly correlated with 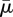 than 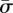. The lack of a significant trend for organic compound types (other than lignin) is also surprising, as we would expect water soluble carbohydrates to affect faster decomposition time scales, and cellulose to also play a role in dynamics.

Latitude, used as a proxy for the variability in seasonal temperature, does not show a correlation with 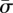, indicating that temporal fluctuations in temperature do not contribute to the rate heterogeneity. A comparison of average monthly temperature and precipitation data with 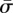 also supports this finding. This result provides further evidence that rate heterogeneity is set by non-climatic factors, and that climate scales the time scale of both labile and refractory processes roughly equally.

## 5. Scaling up to the carbon cycle

The heterogeneity of decomposition rates has strong implications for the dynamic properties of carbon stocks. The derivative of equation (2.1) at *t* = 0 reveals that5.1
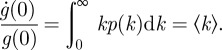


Equation (5.1) states that the effective initial rate of decay is the mean rate constant [31] because all components are initially present. When *p*(*k*) is lognormal,5.2
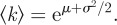


The mean 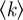 is exponentially greater than the median e^*μ*^ because of the heavy tail of *p*(*k*). A similar amplification acts to exponentially increase the turnover time *τ* to values much greater than 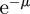. Using equation (4.1) and assuming *p*(*k*) is lognormal, one finds5.3
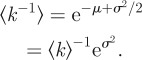
These relations show that rate heterogeneity has a profound effect: 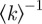 underestimates *τ* by a factor that grows exponentially with the variance *σ*^2^. As the distribution widens, fast rate-constants weigh heavily on the calculation of 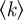, whereas slower rate-constants set the mean residence time 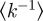. The upshot is that both the size of organic carbon stocks (proportional to *τ* in the steady state) and the time scale of the transient response to a disturbance (also related to *τ*) grow exponentially with the heterogeneity *σ*^2^ of rates. These effects are a consequence of the heavy tail of the lognormal distribution.

**Figure 3.**
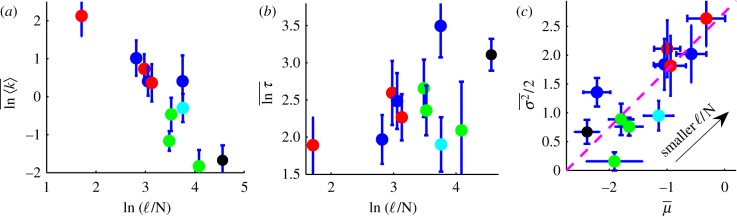
The effect of composition on the initial decomposition rate 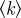 and the turnover time *τ*. The colour of data points indicates the tissue type: roots (blue), leaves (red), needles (green), wood (black) and wheat (cyan). (*a*) 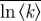 versus the initial lignin-to-nitrogen ratio ℓ/N exhibits a strong negative correlation (*r*_s_ = −0.85, *p* = 0.002, *n* = 11). (*b*) Turnover time 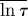 versus ℓ/N shows no significant correlation (*r*_s_ = 0.36, *p* = 0.27, *n* = 11). (*c*) 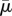 and 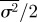 for each litter type are significantly correlated (*r*_s_ = 0.85, *p* = 0.002, *n* = 11) The dashed line represents both a constant turnover time 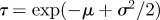 and, by inspection of figure 2*b*,*d*, the direction of changing ℓ/N. Data points represent 11 different litter types averaged over at least four different locations.

We calculate 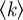 and *τ* for each dataset from our inversion using equations (5.1) and (4.1) and find the average of the log of their values, 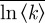 and 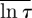, for each litter type. Focusing on the effects of composition, figure 3*a* shows a strong negative correlation between 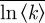 and ℓ/N, whereas figure 3*b* shows no significant correlation between the average order of magnitude of turnover time 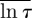 and ℓ/N. Physically, these relations reflect the unequal influence of composition on faster and slower rate constants *k*. Because 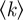 is also the initial decomposition rate, we conclude that the initial ℓ/N exhibits strong control over early decomposition [21,53]. This influence of initial composition is eventually lost, not only at later times [6] but also in the steady state. Mathematically, these observed trends follow from equations (5.2) and (5.3), given that ℓ/N correlates negatively with both 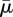 and 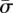 (figure 2*b*,*d*).

We find that leaves, needles and roots on average have roughly the same turnover times: 10 years, 11 years and 14 years, respectively. The geometric mean turnover time of all 191 datasets is 11.5 years, but deviations from this characteristic value appear not to be controlled by initial composition. Recall from §4 that roots may also have uncharacterizably long residence times in certain locations and these are not analysed in figure 3, suggesting a larger departure of root turnover time from needles and leaves. Conditions resulting in extremely persistent root organic matter are unclear (see appendix A.1). Because the turnover time is unaffected by initial nitrogen concentration, we cannot claim that changes in the nitrogen content of the litter (perhaps through changes in nitrogen deposition) will affect the turnover time of plant matter or carbon storage in soils. It is possible that soil composed of the parent material (as opposed to the LIDET transplant study) may show a different relationship between nitrogen and turnover time. Changes in temperature and precipitation on the other hand affect 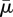 only and therefore do influence turnover time and soil carbon storage. Figure 3*a* additionally shows a separation in initial decay rate among the different litter types, with leaves and roots initially decaying faster than needles. Because the kinetic heterogeneity of roots and needles is wide, one should be especially careful when extrapolating turnover times from short-duration decay experiments associated with these tissue types and other litters with high ℓ/N.

Simple patterns emerge from the relationship between composition, *μ*, *σ*, 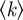 and *τ*. The lack of a trend in figure 3*b*, combined with equation (5.3), suggests that 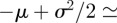 constant, indicating that *μ* and *σ*^2^ may be positively correlated under a compositional change. Figure 3*c* shows that *σ*^2^ is indeed correlated with 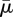 across different litter types. Moreover, the compositional parameter ℓ/N changes the values of 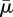 and 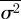 roughly along a line of constant turnover time, as expected when 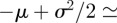 constant. Figure 3*c* also concisely portrays the partitioning of different tissue types in parameter space; needles and wood are characterized by low *μ* and *σ*, leaves by high *μ* and *σ* and roots by a range of *μ* and high *σ*.

The patterns observed in figure 3*a*–*c* suggest the following physical interpretation: initial litter composition tends to change the faster rates in the continuum, which affect both *μ* and *σ*. The slower rates associated with a long-term behaviour and turnover time are less related to initial litter chemistry and are more likely to be determined by soil and microbial community properties. Therefore, during the later stages of litter decay, continued transformation to soil organic matter and its subsequent decay are less a function of the parent material and more a function of semi-transformed compounds and its local interaction with soil [6]. Furthermore, early degradation may be nutrient-limited and depend on the nutrient content of the litter, whereas the slower paced degradation of more recalcitrant materials may be sustained by immobilization of nutrients from the surrounding soil. The departure of roots from the trend in figure 3*c*, specifically the relative constancy of *σ* under a change in *μ*, suggests that the effect of initial composition may persist during root decay or decomposition below ground, influencing the rates of slower processes as well.

## 6. Conclusion

**Figure 4.**
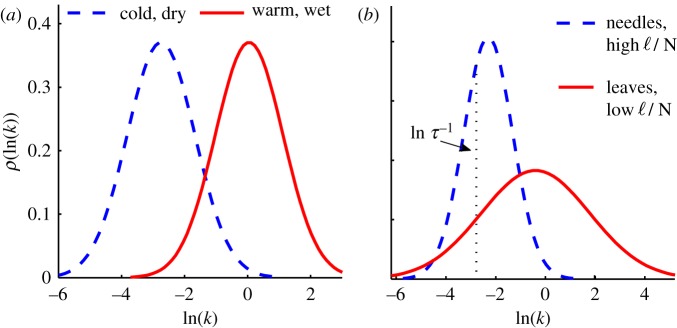
Lognormal distributions *ρ*(ln *k*) associated with different climates and plant-matter compositions. (*a*) Environmental differences tend to shift the distribution along the ln *k* axis. Both distributions have a value of *σ* corresponding to the mean of the data in figure 2*c*. The lower value of *μ* of the (blue) dashed distribution is consistent with values found in colder, drier climates; the higher value of *μ* (solid red distribution) is characteristic of warmer, wetter climates. (*b*) Faster rates are more sensitive to compositional change, e.g. changing the lignin-to-nitrogen ratio ℓ/N, than slower rates. The dashed blue distribution has values of *μ* and *σ* consistent with distributions associated on average with needles or high ℓ/N; the solid red distribution is characteristic on average of leaves or litters with lower ℓ/N. Values of *μ* and *σ* are taken from the dashed line in figure 3*c*; thus both distributions result in the same turnover time *τ*.

**Figure 5.**
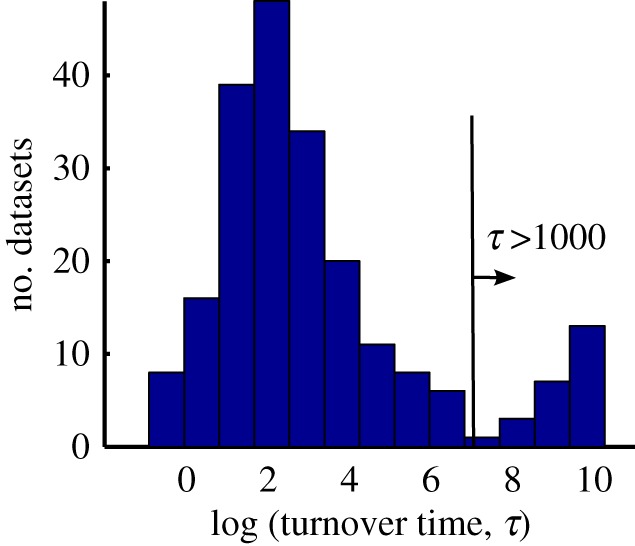
A Histogram of turnover times of 215 LIDET datasets. The vertical black line has a turnover time of 1000 years and indicates a clear separation between a main cluster of datasets, and the beginning of a tail that contains extremely long turnover times. We eliminate those datasets to the right of the black line.

Figure 4 depicts our main findings: (i) decomposition rates are distributed lognormally; (ii) environmental change acts as a catalyst that scales all rates similarly, consistent with the models (such as CENTURY) that assign the same temperature and moisture sensitivity across all pools of organic matter; and (iii) faster processes are more sensitive to litter composition (e.g. ℓ/N, tissue type) than slower processes. The first result, made possible by inverting equation (2.1), identifies the structure of the kinetic heterogeneity associated with decomposition. The second addresses an ongoing debate concerning the temperature sensitivity of decomposition at different time scales [21,55]. The third result identifies a control for the dispersion of decomposition time scales and shows why composition affects initial decay without changing the turnover time. Each conclusion is separate and independent of the others.

Ecosystem models are often coupled with global circulation models [14,57–61] in order to provide an insight into the climate system. Incorporation of lognormally distributed decay rates in popular ecosystem models and use of the lognormal to precisely predict carbon turnover and storage would require careful parametrizations [6,14,16,54] between the lognormal parameters *μ* and *σ* and climatic, soil and compositional parameters. We have provided a first approach for quantifying these relations. However, a more detailed analysis incorporating known mechanisms [6] is required to provide a more comprehensive picture.

We note that the wide range of conditions under which lognormal rates are expected suggests that our results are general, applicable to other degradation processes in natural environments. Evidence of this generality is seen in the decay of marine sedimentary organic matter, which is well described by the quantitatively similar log-uniform distribution [28]. The ubiquity of lognormally distributed degradation rates suggests that a focus on factors that affect rate heterogeneity, rather than specific rates themselves, will lead to a greater understanding—and improved predictions [6,13]—of the ways in which the carbon cycle interacts with climate.

## Figures and Tables

**Figure 6.**
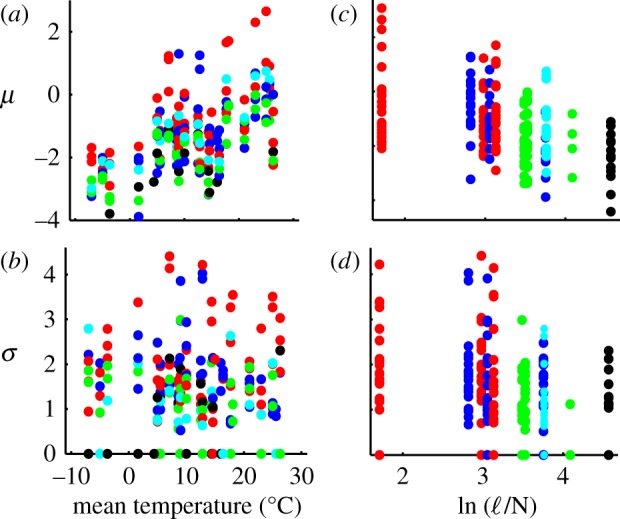
Plots of the unaveraged lognormal parameters *μ* and *σ* versus experimental variables temperature and ℓ/N. All 191 datasets are shown in each figure. Colours indicate tissue types. Roots (blue), leaves (red), needles (green), wood (black) and wheat (cyan). (*a*) *μ* versus mean annual temperature. (*b*) *μ* versus ℓ/N. (*c*) *σ* versus mean annual temperature. (*d*) *σ* versus ℓ/N. Comparison with figure 2 of the main text shows similar trends.

**Figure 7.**
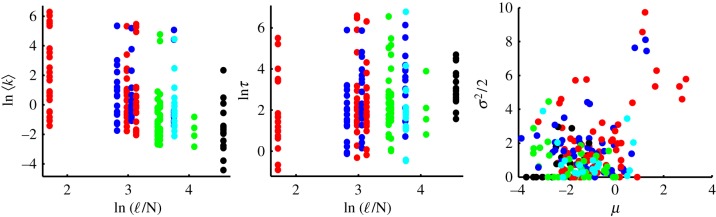
Effect of composition on the unaveraged mean decomposition rate 〈*k*〉 and turnover time *τ*. All 191 datasets are shown in each figure. Colours indicate tissue types. Roots (blue), leaves (red), needles (green), wood (black) and wheat (cyan). (*a*) 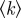 versus ℓ/N. (*b*) *τ* versus ℓ/N. (*c*) *σ*^2^ versus *μ*. Comparison with figure 3 of the main text shows similar trends.

## Appendix A

A.1. Data screening

Our analysis uses litter bag data from the LIDET study [12,21–23]. An important measurement taken during the study is the fraction of original ash-free mass remaining after a given type of plant matter decomposes for a set amount of time in a particular location. Each measurement represents the average of up to four replicates at each site, litter type and removal time. Litter bags were typically collected and analysed each year for up to 10 years, except for bags at tropical and sub-tropical sites, which were more frequently collected at three to six month intervals.

We call a data point the average mass fraction remaining of all replicates of a given plant-matter type, site and duration. If a data point consists of only one litter bag (no replicates), we average that litter bag with the litter bags of the temporally nearest data point (typically 1 year before or 1 year after). We call the time series of data points  associated with a given combination of site and litter type a *dataset.* Before analysis, we subject the LIDET datasets to a series of four tests.

1. First, we consider only the 234 LIDET datasets that each contained at least five data points (not including time zero) with at least one replicate each.
2. The datasets are subjected to a Mann–Kendall test of trend [62] to check for a significant trend of mass loss between the time of the first data point (not time zero) and the final data point. According to this criterion, seven datasets have no significant decay over this duration and are therefore eliminated from further consideration.
3. The remaining datasets are then checked against the assumption that mass loss is described by the superposition of exponential decays given in equation (2.1) of the main text and equation (A 1) given below. To be consistent with the superposition, any decay  must be completely monotone, meaning that all even derivatives of  must be ≥0 and all odd derivatives must be ≤0 (see §XIII.4 of Feller [40]). Because, the Mann–Kendall trend test identifies significant decreasing trends, we additionally check only the average sign of the second derivative. To do so, we fit a quadratic function *a + bt + ct*^2^ to  and eliminate the nine datasets for which *c* < 0.
4. Of the remaining 218 datasets, three more datasets are eliminated because the inversion algorithm cannot identify a solution *p*(*k*) satisfying  and  without having a significant trend in the residual error. The combined application of these four criteria leaves 215 datasets for analysis. As discussed in the text, we find that the average *ρ*(ln *k*) determined from the inversion of these 215 datasets appears lognormal.

Before identifying trends in the parameters *μ* and *σ*, we check their values for outliers. Outliers are identified by checking the turnover times of each dataset by substituting the inversion into using equation (4.1). While 155 out of these 215 datasets had turnover times less than 50 years, a few datasets were characterized by turnover times extremely large for litter decay, over 1000 years. As shown in figure 5, a histogram of turnover times identified two clusters of datasets: a main cluster of 191 datasets having turnover times less than 1000 years, and 24 datasets having turnover times greater than 1000 years. For the remaining analysis of the parameters *μ* and *σ*, we do not analyse the outlying cluster of 24 datasets with *τ* > 1000 for the reasons discussed in the main text. Inclusion of these datasets in our analysis adds noise to our estimates of  and  but does not change general trends.

*A.1.1. Trends in data not well described by a superposition of exponential decays.* We present the datasets that do not appear well described by equation (2.1) in table 2 and find that there are noticeable patterns.

The datasets flagged by test 2 (negligible mass loss) and test 5 (turnover time) are associated with slow degradation and the persistence of organic carbon. Table 2 shows that these datasets are predominantly associated with the decay of *roots* located at the sites CPR, HFR, VCR, NIN, NWT, ARC and BSF. While it is known that roots can be highly recalcitrant [6], it is unclear from this data what controls the persistence of roots; there appear to be no common patterns among the associated sites; they vary highly in climatic parameters and biome type. Although all three types of roots (ANGE, DRGL, PIEL) were also planted at the sites AND, CDR, CWT, HBR, KNZ, LBS, LUQ, LVW, OLY, SMR and UFL, these sites do not contain a single dataset showing extremely slow root decay. There also appears to be no common patterns among these sites.

**Table 2.** Datasets that were flagged by tests 2–5. Datasets numbered 1–19 were not considered in both the inversion and analysis of  and . Datasets 20–43 were included in the inversion but not considered for the analysis of  and . The second column states the code for the location of the dataset, as described in table E1 of the electronic supplementary material. The third column states the substrate code for the dataset as explained in the table footer. The fourth column states the type of tissue used in each experiment, either needle, leaf, root, wood or wheat. The final column states the reason why each dataset is considered not well described by a superposition of rates, as described in the text of this section. The species and common names associated with each code is as follows; ANGE: Andropogon gerardii (Big blue stem), PIEL: Pinus elliottii (Slash pine), THPL: Thuja plicata (Western red cedar), TRAE: Triticum aestivum (Wheat), PIRE: Pinus resinosa (Red pine), QUPR: Quercus prinus (Chestnut oak), GOBA: Gonystylus bananus (Ramin), ACSA: Acer saccharum (Sugar maple), DRGL: Drypetes glauca (Asolillo). *τ* has units [yr]. Descriptions of the LIDET sites can be found in the ESM Table E1.

	site	substrate	tissue	reason
1	CPR	ANGE	root	insignificant mass loss
2	HFR	ANGE	root	insignificant mass loss
3	VCR	ANGE	root	insignificant mass loss
4	NIN	ANGE	root	insignificant mass loss
5	NIN	PIEL	root	insignificant mass loss
6	NWT	PIEL	root	insignificant mass loss
7	SMR	THPL	needle	insignificant mass loss
8	CPR	TRAE	wheat	*g*(*t*) is concave down
9	GSF	TRAE	wheat	*g*(*t*) is concave down
10	GSF	DRGL	root	*g*(*t*) is concave down
11	GSF	PIRE	root	*g*(*t*) is concave down
12	JRN	TRAE	wheat	*g*(*t*) is concave down
13	JRN	PIRE	needle	*g*(*t*) is concave down
14	JRN	THPL	needle	*g*(*t*) is concave down
15	SMR	QUPR	leaf	*g*(*t*) is concave down
16	LUQ	GOBA	wood	*g*(*t*) is concave down
17	BCI	GOBA	wood	trend in residual error
18	BNZ	GOBA	wood	trend in residual error
19	LBS	GOBA	wood	trend in residual error
20	ARC	DRGL	root	*τ* = 2 × 10^4^
21	ARC	PIEL	root	*τ* = 2 × 10^4^
22	BNZ	PIEL	root	*τ* = 8 × 10^3^
23	BSF	ANGE	root	*τ* = 9 × 10^3^
24	BSF	DRGL	root	*τ* = 1 × 10^4^
25	BSF	QUPR	leaf	*τ* = 3 × 10^3^
26	CPR	GOBA	wood	*τ* = 8 × 10^3^
27	CPR	PIEL	root	*τ* = 2 × 10^4^
28	GSF	THPL	needle	*τ* = 1 × 10^4^
29	HFR	PIEL	root	*τ* = 2 × 10^3^
30	JRN	PIEL	root	*τ* = 2 × 10^4^
31	LVW	ACSA	leaf	*τ* = 1 × 10^4^
32	LVW	PIEL	needle	*τ* = 1 × 10^4^
33	LVW	QUPR	leaf	*τ* = 1 × 10^4^
34	NIN	DRGL	root	*τ* = 1 × 10^4^
35	NWT	ANGE	root	*τ* = 3 × 10^3^
36	NWT	DRGL	root	*τ* = 1 × 10^4^
37	SEV	PIEL	root	*τ* = 2 × 10^4^
38	SMR	GOBA	wood	*τ* = 1 × 10^4^
39	SMR	ASCA	leaf	*τ* = 1 × 10^4^
40	UFL	GOBA	wood	*τ* = 3 × 10^3^
41	VCR	DRGL	root	*τ* = 1 × 10^4^
42	VCR	GOBA	wood	*τ* = 3 × 10^4^
43	VCR	PIEL	root	*τ* = 2 × 10^4^

As discussed in the main text, the datasets filtered by tests 3 (negative curvature) or 4 (trend in residual error) both identify faster than exponential decays. These were consistently located at sites that had low precipitation or were wood, indicating that decay in dry conditions and decay of wood may not always be substrate-limited.

A.2. Regularized inversion

As described in the main text, we assume that a superposition of exponential decays

A1

describes a typical decay experiment, where  is the mass fraction remaining at time *t* and *p*(*k*) is the initial probability distribution of first-order decay rates *k*. Mathematically,  is the Laplace transform of a given rate distribution *p*(*k*). As mentioned in the main text, distributions *p*(*k*) have been hypothesized and evaluated by inserting them into equation (A 1) and comparing the resulting  with data [27,28]. In principle, we could calculate the inverse Laplace transform of  exactly to determine *p*(*k*). However, the inverse Laplace transform is ill-posed [44,63], meaning that small changes in  can result in large changes in *p*(*k*), effectively resulting in non-unique solutions. We address this problem as follows.

First, we transform the probability density function *p*(*k*) to , where *x* = ln *k*:

A2

The Laplace transform (A 1) then becomes

A3

To solve for *ρ*(ln *k*) from a given set of data , we discretize equation (A 3) as

A4

where  is a vector of data points  of length *m*,  is the discretized Laplace transform operator of size  and  is the solution vector of length *n* associated with *n* prescribed intervals of ln *k*.

Because the system is both constrained and under-determined (), we seek the  that minimizes the norm of the residual error. In other words, we find the distribution *ρ* that solves

A5

subject to the non-negativity constraint

A6

and the constraint that *ρ*(ln *k*) integrates to unity:

A7

This constrained inversion of the Laplace transform, however, remains ill-posed.

Regularization techniques are commonly used to solve under-determined and ill-posed inverse problems [44,45,63]. These techniques typically work by attempting to find the ‘simplest’ solution that fits the data signal but not the noise. Here, we use a Tikhonov regularization technique [44,45,63], which favours smooth solutions by minimizing both the residual error and a quantitative measure of the complexity of *ρ*(ln *k*). Solution complexity is commonly associated with the roughness or the intensity of fluctuations present in the solution. Here, we measure roughness by the squared norm of the first derivative of the solution vector, i.e.

A8

The problem then becomes

A9

where  is the bi-diagonal first derivative operator (i.e. the discretization of equation (A 8)) and *λ* is the regularization parameter, which controls the relative weights of the solution roughness and the residual error norm.

The method proceeds by identifying the value of *λ* that sets an optimal balance between the residual error  and the roughness . A common approach is to use an L-curve technique [44,63]. An L-curve is generated by parametrically varying *λ* and solving equation (A 9) for *ρ*(ln *k*), obtaining values for the residual error norm  and the roughness norm  for each value of *λ*. The parametric relationship of  versus  typically has the shape of an ‘L’. Although each point on the L-curve may be considered an optimal solution for a certain value of *λ*, the corner of this curve is associated with the regularized solution to the inverse problem. The corner represents the value of *λ* for which increasing *λ* strongly increases solution roughness, while providing little reduction in residual norm, and also for which decreasing *λ* greatly sacrifices residual norm with little reduction in roughness.

Before choosing the corner, we check the solution with *λ* = 0 in case it is just a single delta function, indicating that only one rate is present during decay. If greater than 0.99 of the mass fraction is contained within one rate, we conclude that the simplest solution is an exponential decay having a single rate constant. This occurs for 33 of the datasets.

A.3. Unaveraged parameter analysis

We present here the unaveraged data shown in figures 2 and 3 of the main text. Figure 6 shows how the values of *μ* and *σ* for the 191 LIDET datasets vary with temperature and the lignin-to-nitrogen ratio ℓ/N. Figure 7*a,b* show the unaveraged variation of  and *τ* with ℓ/N. Figure 7*c* shows the unaveraged plot of *σ*^2^ versus *μ*. While there is much scatter among the data, the general trends remain the same as in figures 2 and 3 of the main text.
